# Supplementary material for: Integrated analysis of differentially expressed profiles and construction of a competing endogenous long non-coding RNA network in renal cell carcinoma
Source: PeerJ. 2018 Jul 17;6:e5124. doi: 10.7717/peerj.5124 (PMC6054097; doi:10.7717/peerj.5124)
Supplement: Table S1 [file peerj-06-5124-s001.docx]

**Supplementary Table 1.** Top ten of up-regulated and down-regulated intersection lncRNAs.

| lncRNA | Gene ID | Expression change (Tumor vs control) |
| --- | --- | --- |
| LINC00887 | 100131551 | Up-regulation |
| FER1L4 | 80307 | Up-regulation |
| DGCR5 | 26220 | Up-regulation |
| PVT1 | 5820 | Up-regulation |
| LINC01587 | 10141 | Up-regulation |
| DGCR9 | 25787 | Up-regulation |
| HSPA7 | 3311 | Up-regulation |
| CXCR2P1 | 3580 | Up-regulation |
| DGCR10 | 26222 | Up-regulation |
| DUSP5P1 | 574029 | Up-regulation |
| LOC284578 | 284578 | Down-regulation |
| LINC00982 | 440556 | Down-regulation |
| GATA3-AS1 | 399717 | Down-regulation |
| LINC00473 | 90632 | Down-regulation |
| LINC00284 | 121838 | Down-regulation |
| SSTR5-AS1 | 146336 | Down-regulation |
| TDGF1P3 | 6998 | Down-regulation |
| PART1 | 25859 | Down-regulation |
| LINC01018 | 255167 | Down-regulation |
| LINC00461 | 645323 | Down-regulation |
