# Supplementary material for: Integrated analysis of differentially expressed profiles and construction of a competing endogenous long non-coding RNA network in renal cell carcinoma
Source: PeerJ. 2018 Jul 17;6:e5124. doi: 10.7717/peerj.5124 (PMC6054097; doi:10.7717/peerj.5124)
Supplement: Table S2 [file peerj-06-5124-s002.docx]

**Supplementary Table 2.** Top 20 GO gene sets correlate with up-regulated mRNAs by GSEA

| GO Name | SIZE | ES | NES | NOM p-val | FDR q-val |
| --- | --- | --- | --- | --- | --- |
| GO_REGULATION_OF_IMMUNE_RESPONSE | 187 | 0.52 | 3.79 | <0.001 | <0.001 |
| GO_IMMUNE_RESPONSE | 261 | 0.49 | 3.76 | <0.001 | <0.001 |
| GO_IMMUNE_SYSTEM_PROCESS | 391 | 0.44 | 3.56 | <0.001 | <0.001 |
| GO_POSITIVE_REGULATION_OF_IMMUNE_SYSTEM_PROCESS | 195 | 0.48 | 3.55 | <0.001 | <0.001 |
| GO_POSITIVE_REGULATION_OF_IMMUNE_RESPONSE | 119 | 0.53 | 3.54 | <0.001 | <0.001 |
| GO_REGULATION_OF_IMMUNE_SYSTEM_PROCESS | 291 | 0.44 | 3.48 | <0.001 | <0.001 |
| GO_LEUKOCYTE_ACTIVATION | 113 | 0.5 | 3.28 | <0.001 | <0.001 |
| GO_IMMUNE_RESPONSE_REGULATING_CELL_SURFACE_RECEPTOR_SIGNALING_PATHWAY | 64 | 0.56 | 3.26 | <0.001 | <0.001 |
| GO_DEFENSE_RESPONSE | 267 | 0.41 | 3.21 | <0.001 | <0.001 |
| GO_LYMPHOCYTE_ACTIVATION | 96 | 0.51 | 3.2 | <0.001 | <0.001 |
| GO_CELL_ACTIVATION | 137 | 0.47 | 3.18 | <0.001 | <0.001 |
| GO_ADAPTIVE_IMMUNE_RESPONSE | 80 | 0.52 | 3.17 | <0.001 | <0.001 |
| GO_INNATE_IMMUNE_RESPONSE | 119 | 0.47 | 3.17 | <0.001 | <0.001 |
| GO_POSITIVE_REGULATION_OF_CELL_ACTIVATION | 89 | 0.51 | 3.16 | <0.001 | <0.001 |
| GO_IMMUNE_EFFECTOR_PROCESS | 95 | 0.49 | 3.15 | <0.001 | <0.001 |
| GO_REGULATION_OF_CELL_ACTIVATION | 133 | 0.45 | 3.13 | <0.001 | <0.001 |
| GO_ACTIVATION_OF_IMMUNE_RESPONSE | 87 | 0.51 | 3.1 | <0.001 | <0.001 |
| GO_REGULATION_OF_CYTOKINE_PRODUCTION | 107 | 0.46 | 3.02 | <0.001 | <0.001 |
| GO_POSITIVE_REGULATION_OF_CELL_CELL_ADHESION | 77 | 0.49 | 2.97 | <0.001 | <0.001 |
| GO_LEUKOCYTE_CELL_CELL_ADHESION | 69 | 0.51 | 2.96 | <0.001 | <0.001 |
