# Supplementary material for: Integrated analysis of differentially expressed profiles and construction of a competing endogenous long non-coding RNA network in renal cell carcinoma
Source: PeerJ. 2018 Jul 17;6:e5124. doi: 10.7717/peerj.5124 (PMC6054097; doi:10.7717/peerj.5124)
Supplement: Table S3 [file peerj-06-5124-s003.docx]

**Supplementary Table 3.** Top 20 GO gene sets correlate with down-regulated mRNAs by GSEA

| GO Name | SIZE | ES | NES | NOM p-val | FDR q-val |
| --- | --- | --- | --- | --- | --- |
| GO_UROGENITAL_SYSTEM_DEVELOPMENT | 65 | -0.55 | -2.87 | <0.001 | <0.001 |
| GO_KIDNEY_EPITHELIUM_DEVELOPMENT | 34 | -0.62 | -2.75 | <0.001 | <0.001 |
| GO_NEPHRON_DEVELOPMENT | 32 | -0.61 | -2.66 | <0.001 | <0.001 |
| GO_METANEPHROS_DEVELOPMENT | 23 | -0.66 | -2.61 | <0.001 | <0.001 |
| GO_NEPHRON_EPITHELIUM_DEVELOPMENT | 27 | -0.62 | -2.56 | <0.001 | <0.001 |
| GO_CARDIAC_CHAMBER_MORPHOGENESIS | 16 | -0.71 | -2.51 | <0.001 | <0.001 |
| GO_MESONEPHROS_DEVELOPMENT | 22 | -0.65 | -2.5 | <0.001 | <0.001 |
| GO_RENAL_TUBULE_DEVELOPMENT | 23 | -0.61 | -2.38 | <0.001 | 0.001 |
| GO_SODIUM_ION_HOMEOSTASIS | 11 | -0.75 | -2.37 | <0.001 | 0.001 |
| GO_CARDIAC_CHAMBER_DEVELOPMENT | 20 | -0.62 | -2.36 | <0.001 | 0.001 |
| GO_KIDNEY_MORPHOGENESIS | 24 | -0.59 | -2.34 | <0.001 | 0.002 |
| GO_REGIONALIZATION | 44 | -0.49 | -2.3 | <0.001 | 0.003 |
| GO_TISSUE_MORPHOGENESIS | 81 | -0.42 | -2.3 | <0.001 | 0.003 |
| GO_ACTOMYOSIN_STRUCTURE_ORGANIZATION | 10 | -0.76 | -2.26 | <0.001 | 0.004 |
| GO_ORGANIC_ACID_CATABOLIC_PROCESS | 31 | -0.52 | -2.24 | <0.001 | 0.005 |
| GO_PATTERN_SPECIFICATION_PROCESS | 56 | -0.44 | -2.22 | <0.001 | 0.005 |
| GO_CELLULAR_RESPONSE_TO_STEROID_HORMONE_STIMULUS | 29 | -0.52 | -2.2 | <0.001 | 0.007 |
| GO_TUBE_DEVELOPMENT | 93 | -0.39 | -2.19 | <0.001 | 0.008 |
| GO_MONOCARBOXYLIC_ACID_CATABOLIC_PROCESS | 16 | -0.61 | -2.17 | <0.001 | 0.009 |
| GO_CARDIAC_VENTRICLE_MORPHOGENESIS | 11 | -0.7 | -2.16 | 0.004 | 0.009 |
