# Supplementary material for: Integrated analysis of differentially expressed profiles and construction of a competing endogenous long non-coding RNA network in renal cell carcinoma
Source: PeerJ. 2018 Jul 17;6:e5124. doi: 10.7717/peerj.5124 (PMC6054097; doi:10.7717/peerj.5124)
Supplement: Table S4 [file peerj-06-5124-s004.docx]

**Supplementary Table 4.** Top 20 KEGG gene sets correlate with up-regulated mRNAs by GSEA

| Pathway Name | SIZE | ES | NES | NOM p-val | FDR q-val |
| --- | --- | --- | --- | --- | --- |
| KEGG_NATURAL_KILLER_CELL_MEDIATED_CYTOTOXICITY | 33 | 0.6 | 2.87 | <0.001 | <0.001 |
| KEGG_AUTOIMMUNE_THYROID_DISEASE | 19 | 0.62 | 2.49 | <0.001 | <0.001 |
| KEGG_CYTOKINE_CYTOKINE_RECEPTOR_INTERACTION | 71 | 0.41 | 2.43 | <0.001 | <0.001 |
| KEGG_LEISHMANIA_INFECTION | 19 | 0.6 | 2.39 | <0.001 | <0.001 |
| KEGG_GRAFT_VERSUS_HOST_DISEASE | 17 | 0.62 | 2.39 | <0.001 | <0.001 |
| KEGG_SYSTEMIC_LUPUS_ERYTHEMATOSUS | 26 | 0.54 | 2.39 | <0.001 | <0.001 |
| KEGG_HEMATOPOIETIC_CELL_LINEAGE | 28 | 0.53 | 2.38 | <0.001 | <0.001 |
| KEGG_CHEMOKINE_SIGNALING_PATHWAY | 37 | 0.49 | 2.37 | <0.001 | <0.001 |
| KEGG_TOLL_LIKE_RECEPTOR_SIGNALING_PATHWAY | 13 | 0.65 | 2.31 | <0.001 | <0.001 |
| KEGG_ANTIGEN_PROCESSING_AND_PRESENTATION | 22 | 0.56 | 2.26 | <0.001 | <0.001 |
| KEGG_INTESTINAL_IMMUNE_NETWORK_FOR_IGA_PRODUCTION | 17 | 0.58 | 2.24 | <0.001 | <0.001 |
| KEGG_ALLOGRAFT_REJECTION | 17 | 0.59 | 2.24 | <0.001 | <0.001 |
| KEGG_TYPE_I_DIABETES_MELLITUS | 17 | 0.59 | 2.21 | 0.002 | 0.001 |
| KEGG_PRIMARY_IMMUNODEFICIENCY | 20 | 0.52 | 2.11 | 0.002 | 0.002 |
| KEGG_VIRAL_MYOCARDITIS | 22 | 0.5 | 2.1 | 0.005 | 0.002 |
| KEGG_NOD_LIKE_RECEPTOR_SIGNALING_PATHWAY | 10 | 0.66 | 2.03 | 0.002 | 0.004 |
| KEGG_FC_EPSILON_RI_SIGNALING_PATHWAY | 12 | 0.59 | 1.96 | 0.004 | 0.006 |
| KEGG_CELL_CYCLE | 17 | 0.47 | 1.78 | 0.009 | 0.024 |
| KEGG_ASTHMA | 13 | 0.51 | 1.77 | 0.021 | 0.024 |
| KEGG_B_CELL_RECEPTOR_SIGNALING_PATHWAY | 12 | 0.52 | 1.77 | 0.018 | 0.024 |
