# Supplementary material for: Integrated analysis of differentially expressed profiles and construction of a competing endogenous long non-coding RNA network in renal cell carcinoma
Source: PeerJ. 2018 Jul 17;6:e5124. doi: 10.7717/peerj.5124 (PMC6054097; doi:10.7717/peerj.5124)
Supplement: Table S5 [file peerj-06-5124-s005.docx]

**Supplementary Table 5.** Top 20 KEGG gene sets correlate with down-regulated mRNAs by GSEA

| Pathway Name | SIZE | ES | NES | NOM p-val | FDR q-val |
| --- | --- | --- | --- | --- | --- |
| KEGG_ALDOSTERONE_REGULATED_SODIUM_REABSORPTION | 10 | -0.76 | -2.27 | <0.001 | <0.001 |
| KEGG_TIGHT_JUNCTION | 20 | -0.56 | -2.09 | <0.001 | 0.006 |
| KEGG_ERBB_SIGNALING_PATHWAY | 11 | -0.52 | -1.62 | 0.046 | 0.267 |
| KEGG_METABOLISM_OF_XENOBIOTICS_BY_CYTOCHROME_P450 | 14 | -0.45 | -1.53 | 0.046 | 0.35 |
| KEGG_WNT_SIGNALING_PATHWAY | 13 | -0.47 | -1.52 | 0.059 | 0.292 |
| KEGG_DRUG_METABOLISM_CYTOCHROME_P450 | 14 | -0.45 | -1.51 | 0.052 | 0.261 |
| KEGG_MAPK_SIGNALING_PATHWAY | 28 | -0.34 | -1.41 | 0.078 | 0.376 |
| KEGG_RETINOL_METABOLISM | 16 | -0.37 | -1.32 | 0.148 | 0.498 |
| KEGG_GAP_JUNCTION | 11 | -0.42 | -1.31 | 0.177 | 0.455 |
| KEGG_ARRHYTHMOGENIC_RIGHT_VENTRICULAR_CARDIOMYOPATHY_ARVC | 10 | -0.41 | -1.22 | 0.244 | 0.589 |
| KEGG_AXON_GUIDANCE | 24 | -0.3 | -1.19 | 0.247 | 0.608 |
| KEGG_HYPERTROPHIC_CARDIOMYOPATHY_HCM | 11 | -0.37 | -1.15 | 0.289 | 0.634 |
| KEGG_COMPLEMENT_AND_COAGULATION_CASCADES | 23 | -0.29 | -1.15 | 0.287 | 0.586 |
| KEGG_STEROID_HORMONE_BIOSYNTHESIS | 11 | -0.36 | -1.12 | 0.306 | 0.615 |
| KEGG_REGULATION_OF_ACTIN_CYTOSKELETON | 32 | -0.26 | -1.11 | 0.317 | 0.587 |
| KEGG_ARGININE_AND_PROLINE_METABOLISM | 13 | -0.33 | -1.08 | 0.367 | 0.599 |
| KEGG_ENDOCYTOSIS | 28 | -0.25 | -1.04 | 0.413 | 0.638 |
| KEGG_MELANOGENESIS | 13 | -0.3 | -1 | 0.441 | 0.681 |
| KEGG_DILATED_CARDIOMYOPATHY | 13 | -0.31 | -0.99 | 0.458 | 0.66 |
| KEGG_LYSOSOME | 11 | -0.31 | -0.97 | 0.488 | 0.671 |
