# Supplementary material for: Integrated analysis of differentially expressed profiles and construction of a competing endogenous long non-coding RNA network in renal cell carcinoma
Source: PeerJ. 2018 Jul 17;6:e5124. doi: 10.7717/peerj.5124 (PMC6054097; doi:10.7717/peerj.5124)
Supplement: Table S6 [file peerj-06-5124-s006.docx]

**Supplementary Table 6**. RNAs significantly associated with overall survival in ceRNA network.

| lncRNA | MiRNA | mRNA |
| --- | --- | --- |
| AATBC, ADAM6, ADCY10P1, ADGRE4P, AFAP1-AS1, ALOX12P2, CHKB-CPT1B, COL18A1-AS1, CRYM-AS1, DDX12P, EMBP1, EWSAT1, FAM13A-AS1, FAM182B, FAM225B, FER1L4, FOXD2-AS1, GAS2L1P2, GOLGA6L5P, HERC2P2, HLA-L, HOXA11-AS, INE1, LHFPL3-AS2, LINC00174, LINC00323, LINC00475, LINC00685, LINC00893, LINC01000, ,LINC01001, LOC100129034, LOC100132215, LOC100132287, LOC150776, LOC155060, LOC642846, LOC729603, LPAL2, MALAT1, MBL1P, MIAT, MIR31HG, MIR4435-2HG, MTMR9LP, N4BP2L2-IT2, PDXDC2P, PP14571, PPIEL, RPL13AP3, RRN3P2, SCART1, SMIM10L2B, SNHG3, SSTR5-AS1, TINCR, ,TRHDE-AS1, TSPY26P, UBE2Q2P1, UG0898H09, USP32P1, WT1-AS, ZNF542P | miR-9-5p  miR-21-5p  miR-155-5p  miR-224-5p | AFP, ATP2B2, ATP2B3, BBC3, CCL5, CCNE2, CDH3, COL1A1, CYFIP2, DLG2, E2F2, EIF4EBP1, FANCA, FGF1, GRIN2D, IGSF5, KCNK10, KCNMA1, MYB, NKD1, PPARGC1A, PPP1R1B, RAB3B, RELN, RUNX1, SEMA3D, SEMA3G, SORD, STAT4, THRB, TP73 |
