# Supplementary figures and images for: Integrated analysis of differentially expressed profiles and construction of a competing endogenous long non-coding RNA network in renal cell carcinoma
Source: PeerJ. 2018 Jul 17;6:e5124. doi: 10.7717/peerj.5124 (PMC6054097; doi:10.7717/peerj.5124)

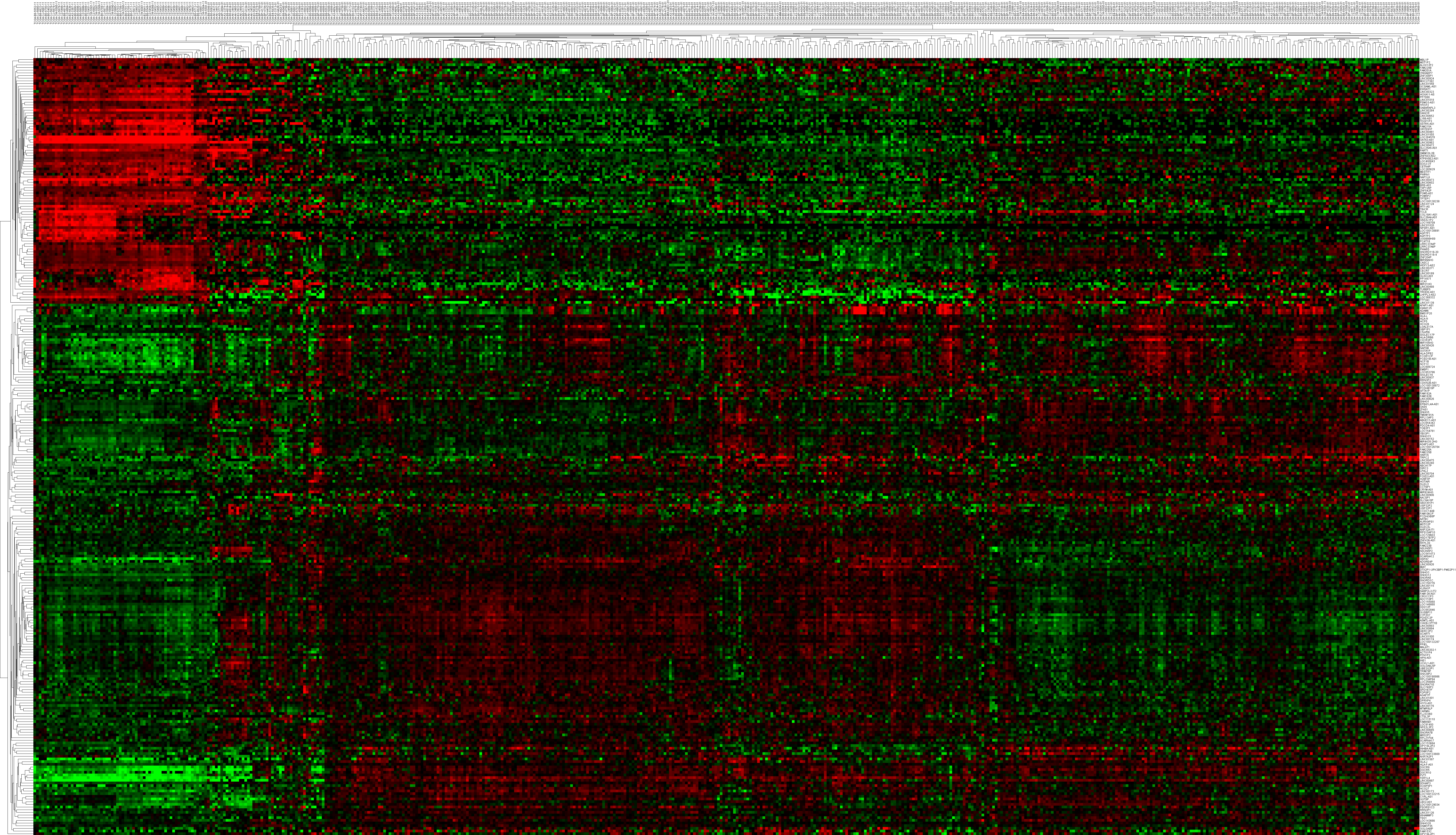

Supplement: Figure S1 — The horizontal axis on top shows the sample names. The right vertical axis displays the names of the lncRNAs, while the left vertical axis represents gene clustering. The expression values are described by a color scale, in which red indicates high expression, while green indicates low expression. [file peerj-06-5124-s007.png]
